# Development and validation of an event-specific detection method for WYN029GmA soybean based on TaqMan qPCR

**DOI:** 10.3389/fpls.2026.1862064

**Published:** 2026-06-12

**Authors:** Hongye Jiang, Hua Zhang, Yuting Miao, Haoqian Wang, Fangqi Fu, Chenyao Wang, Jiake Li, Dan Wang, Xiaodan Jiang, Wei Fu, Junguang He, Hong Chen

**Affiliations:** 1 Development Center of Science and Technology, Ministry of Agriculture and Rural Affairs (MARA), Beijing, China; 2 Changsha Jiemeiao Biotechnology Co., Ltd., Changsha, China

**Keywords:** event-specific detection, qPCR, quantification, validation, WYN029GmA

## Abstract

This study aimed to establish an event-specific detection method for the genetically modified soybean WYN029GmA. WYN029GmA is a glyphosate-resistant transgenic soybean carrying the *mam79 epsps* gene, independently developed in China, and has been granted the China Agricultural Genetically Modified Organism Safety Certificate (Production Application). Based on the junction region between the exogenous insertion sequence and the flanking genomic sequences of WYN029GmA, multiple sets of primers and probes were designed. After screening, WYN029LB-QF4/QR2/QP2 was selected as the optimal primer-probe combination, and the amplified fragment length is 95 bp. The method was systematically evaluated by a series of tests such as specificity, dynamic range, accuracy, and robustness, and its practical applicability was evaluated through an interlaboratory validation involving eight laboratories. The results demonstrated that this method showed good specificity and repeatability across different laboratories, and could meet the technical requirements of qualitative and quantitative detection of WYN029GmA. This method provides reliable technical support for the traceability, safety supervision, and labeling management of WYN029GmA soybean and its derivatives.

## 1 Introduction

With the rapid development of global agricultural biotechnology, genetically modified (GM) crops have become an important means of ensuring food security and improving agricultural production efficiency. In 2024, the global GM crop area increased by 1.9% to 209.8 million hectares, with soybean accounting for half of this area (GM AgbioInvestor Monitor, 2025a). Glyphosate-tolerant transgenic soybeans have become an important part of global agricultural production due to their significant advantages in weed control (Duke and Powles, 2008; Green, 2014). To regulate the cultivation and application of GM crops and protect consumers’ right to know, many countries have issued relevant regulations to require labeling of foods containing GM ingredients (Gruère and Rao, 2007; Wunderlich and Gatto, 2015).

The implementation of global GM labeling policies and safety regulations requires standardized and reliable detection methods (Huang and Pan, 2005; Shin et al., 2023). Among various DNA-based detection techniques, PCR targeting the unique junction regions between the recipient genome and the inserted DNA is widely regarded as the gold standard for GM content identification and quantification due to its highest level of specificity (Holst-Jensen et al., 2003). In particular, TaqMan real-time quantitative PCR (qPCR) has been extensively validated and routinely applied for GM detection, and many methods have been recorded in databases such as GMOMETHODS (Bonfini et al., 2012). Although digital PCR (dPCR) has emerged as a complementary tool enabling absolute quantification without a standard curve and showing comparable or even superior precision in complex matrices (Iwobi et al., 2016; Quan et al., 2018; Cottenet et al., 2019; Bogožalec Košir et al., 2019), qPCR remains the most easily accessible and widely adopted method in routine regulatory testing laboratories (Wissel et al., 2022; Trapmann et al., 2025). However, numerous TaqMan qPCR detection methods have been reported for GM maize and soybean events (Takabatake et al., 2011; Long et al., 2021; Li et al., 2022; Yao et al., 2024; Long et al., 2024; Li et al., 2025; Wu et al., 2026), no such method for WYN029GmA has been reported yet.

WYN029GmA is a novel herbicide-tolerant soybean transformant with *mam79 epsps* gene, developed by Zhejiang Xinan Chemical Industrial Group Co., Ltd. (in brief Wynca). In 2024, it was granted the China Agricultural Genetically Modified Organism Safety Certificate (Production Application). The research team optimized the *am79 epsps* gene based on the codon preference of soybean, and named the modified gene *mam79 epsps*. The *am79 epsps* gene is a novel glyphosate-tolerant gene with Chinese independent intellectual property, cloned from glyphosate-contaminated soil (Lin et al., 2012; Wang et al., 2017). “There are no previous instances of these genes having been granted cultivation or import approval in any other country” (GM AgbioInvestor Monitor, 2025b). The research team inserted the *mam79 epsps* expression cassette into the vector pC3301 and introduced the exogenous sequence into the recipient soybean Tianlong 1 (TL1) via *Agrobacterium*-mediated transformation. After years of experimental screening, the herbicide-tolerant soybean WYN029GmA with excellent agronomic traits was obtained. Therefore, as an independent innovation in the core germplasm resources of biological breeding in China, WYN029GmA urgently requires the establishment of an event-specific detection method.

This study established an event-specific real-time PCR method for detecting and quantifying GM soybean WYN029GmA based on its molecular characterization. We systematically tested key parameters of the method including specificity, sensitivity, dynamic range, limit of detection (LOD), and limit of quantification (LOQ). Furthermore, the performance of the method was validated through an interlaboratory trial involving eight laboratories to assess its practical applicability. The development and application of this method will provide reliable technical support for the traceability, safety supervision, and quantitative identification of WYN029GmA soybean.

## 2 Materials and methods

### 2.1 Plant material

Seeds of homozygous GM soybean WYN029GmA and its non-GM soybean TL1 were provided by Wynca. The six mixed seed powder samples used for specificity testing were from the Development Center of Science and Technology, MARA. These samples were as follows: a mixture of 28 GM corn samples, a mixture of 19 other GM soybean samples, a mixture of eight GM rice samples, a mixture of seven GM cotton samples, a mixture of 10 GM rapeseed samples, and a mixture of non-GM soybean samples. All these samples served as sources of genomic DNA for the purpose of this study.

### 2.2 Sample preparation

Soybean seeds of WYN029GmA and TL1 were sown in a GXZ-450A light incubator under a photoperiod of 16 h light/8 h dark at 25 °C. The fresh leaves after specificity and zygosity tests were frozen in liquid nitrogen and ground using a Retsch MM400 mixer mill. Genomic DNA (gDNA) was isolated from the ground material using the DNeasy Plant Maxi Kit (68163, Qiagen, Germany). The quality and quantity of isolated DNA were estimated by the Quawell Q5000 spectrophotometer. The DNA concentration of WYN029GmA for standard curve preparation was adjusted to 40 ng/µL by dilution. The DNA concentrations of WYN029GmA and TL1 for test samples were each diluted to 25 ng/µL.

All experiments included blank controls and negative controls (TL1). The sample for primer-probe screening and primer-probe concentration determination test was 1% WYN029GmA. The samples of the specificity test included 1% WYN029GmA, 5 different transgenic mixed samples and 1 non-transgenic soybean mixed sample. For LOD testing, five samples with WYN029GmA event copy number ratios of 0.10%, 0.05%, 0.025%, 0.0125%, and 0.0025% (corresponding to 40, 20, 10, 5, and 1 haploid genome copy per PCR reaction, respectively) were tested, each with 10 technical replicates. For the standard curve assay, gDNA from homozygous WYN029GmA was serially diluted at an eight-fold ratio using EASY Dilution (9160, TaKaRa, China) to prepare five calibrators at concentrations of 40.00, 5.00, 0.63, 0.08, and 0.01 ng/µL. For the accuracy test, four test samples with WYN029GmA event copy number ratios of 5%, 3%, 1%, and 0.1% were used; each test sample was set up with three parallel subsamples, each subsample was set up with three technical replicates. For LOQ testing, six samples with WYN029GmA event copy number ratios of 0.125%, 0.10%, 0.075%, 0.05%, 0.025%, and 0.0025% (corresponding to 50, 40, 30, 20, 10, and 1 haploid genome copy per PCR reaction, respectively) were tested, each with 10 technical replicates. Robustness testing included the robustness of LOD and LOQ. LOD robustness was tested twice by replacing operators and instruments, with samples including 1% WYN029GmA and the LOD sample; each sample was tested with 10 technical replicates. LOQ robustness was tested 3 times by replacing operators, instruments, and reagents, using the LOQ sample; each test included 3 parallel subsamples, and each subsample was tested with 3 technical replicates.

The copy number of gDNA was estimated based on the haploid genome size of soybean being 1115 Megabasepairs (Arumuganathan and Earle, 1991), corresponding to a weight of 1.13 pg (Greilhuber and Obermayer, 1997).

### 2.3 Primers and probes design

According to the universal design principles (Rodríguez et al., 2015), event-specific primers and probes for WYN029GmA were designed using Beacon Designer 8 software (PREMIER Biosoft International, Palo Alto, CA). The optimal primer-probe combination was WYN029LB−QF4/QR2/QP2, and its sequences are listed in **Table 1**. *Lectin* was used as a soybean-specific endogenous reference DNA for quantitative analysis. For specific detection of *Lectin*, the primers (*Lectin-*qF: 5’-GCCCTCTACTCCACCCCCA-3’ and *Lectin-*qR: 5’-GCCCATCTGCAAGCCTTTTT-3’) and fluorescent dye-labeled probe (*Lectin-*qP: 5’-AGCTTCGCCGCTTCCTTCAACTTCAC-3’) were used (Kuribara et al., 2002). The oligonucleotide probes were labeled with 5’-FAM (6-carboxyfluorescein) and 3’-BHQ1 (black hole quencher1). The primers and probes were synthesized by Sangon Biotech (Shanghai, China).

**Table 1:** Primer and probe sequences of qPCR for WYN029GmA.

5′ primers and probes	Sequence (5′-3′)	3′ primers and probes	Sequence (5′-3′)
WYN029LB-QF1	TCGTCAAATGTCATCCGTTTA	WYN029RB-QF1	GCGGTGTCATCTATGTTACTAG
WYN029LB-QF2	GACCAAGTATTCAGGAACAGG	WYN029RB-QF2	GAGTCCCGCAATTATACATTTAATAC
WYN029LB-QF3	CATCCGTTTAATTTACTCGCAC	WYN029RB-QR1	TCCCAACAAATAATGAAACAAAGG
WYN029LB-QF4	TACTCGCACTTTTGTTGTTTCAGACC	WYN029RB-QP1	CTCCGCCACCGATGGTTCTAGTTATACTGA
WYN029LB-QR1	GGTCTTCTGAGACTGTATCTTTG		
WYN029LB-QR2	GTGTCGTGCTCCACCATGTTGA		
WYN029LB-QP1	TGTGTCGTGCTCCACCATGTTGAC		
WYN029LB-QP2	CTTGTCATTGAGCCGCCCCTGTTCCT		

### 2.4 Real-time PCR

Three real-time PCR instruments (CFX96, Bio-Rad, USA; StepOnePlus and QuantStudio 3, Applied Biosystems, USA) and three reagent kits (TaqMan™ Gene Expression Master Mix, 4369016, Applied Biosystems; TaqMan™ Fast Advanced Master Mix, 4444557, Applied Biosystems; Premix Ex Taq™ Probe qPCR, RR390A, TaKaRa) were used in this study. Except for primer-probe screening and robustness testing, all other experiments were performed on the CFX96 using TaqMan™ Fast Advanced Master Mix. The total reaction volume was 25 μL, containing 12.5 μL qPCR TaqMan master mix, 400 nM primers, and 200 nM probe, 2 μL DNA template, and sterile distilled water to make up the volume. Except for the standard curve test, the DNA template concentration for all experiments was 25 ng/μL. The reaction program was as follows: initial denaturation at 95 °C for 5 min; 40 cycles of denaturation at 95 °C for 15 s and annealing/extension at 60 °C for 1 min; fluorescence signals were collected during the annealing/extension stage (60 °C). The copy numbers of the WYN029GmA event and the *Lectin* gene in test samples were calculated based on the standard curves and corresponding Cq values, respectively. The GMO content was calculated as the ratio of the WYN029GmA event copy number to the *Lectin* gene copy number.

### 2.5 Interlaboratory method performance study

This experiment was collaboratively conducted by eight genetically modified organism (GMO) testing laboratories. Each laboratory received six tubes of mixed seed powder samples for specificity testing, as well as 18 tubes of genomic samples, including: one tube of TL1 negative control; one tube of WYN029GmA calibrator G1, used to construct a standard curve via serial dilution; one tube of LOD (0.05%) test sample; five blind samples (designated q1–q5), representing WYN029GmA contents of 5%, 3%, 1%, 0.5% and 0.1%, respectively, each consisting of three parallel subsamples. Sample q2 also served as the positive quantitative quality control, q3 as the positive control, and q5 as the LOQ test sample. All samples were prepared at a concentration of 25 ng/µL, except for calibrator G1, which was prepared at 50 ng/µL. A sufficient quantity of each sample was prepared for testing. The samples, together with primers and probes were shipped under refrigerated conditions to each participating laboratory.

## 3 Results and discussion

### 3.1 Design and screening of primers and probes

**Figure 1:**
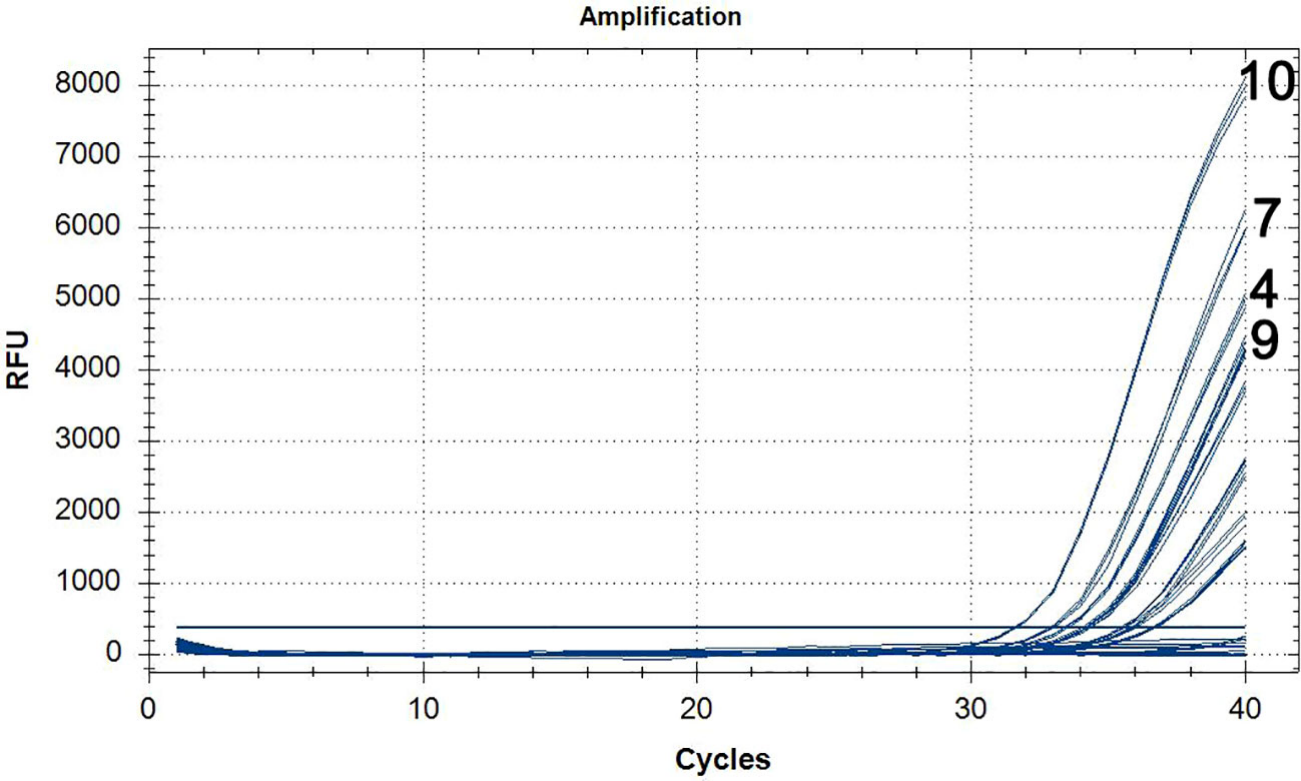
qPCR amplification results of 12 primer-probe combinations of WYN029GmA under a representative condition. Instrument, CFX96; Reagent, TaqMan™ Gene Expression Master Mix. Curves 10, 7, 4, and 9 represent primer-probe combinations 10, 7, 4, and 9, respectively.

A total of 12 primer-probe combinations were designed in this experiment. The sequences of the primers and probes are listed in **Table 1**, and the details of each combination are provided in **Supplementary Table 1**. In the screening test, for each primer-probe combination, negative controls and sample templates were set up with three technical replicates. The experimental results are shown in **Figure 1**, **Supplementary Figure 1**. Combination 9 exhibited the best amplification performance in three instruments and two reagents (**Supplementary Figure 1**), while its performance was slightly worse with one reagent (**Figure 1**). Combination 10 showed relatively stable performance in three instruments and three reagents, with high fluorescence signals, low Cq values and good specificity. Therefore, primer-probe combinations 9 and 10 were preliminarily selected as candidates for further analysis.

### 3.2 Specificity testing and determination of primer-probe combination

**Figure 2:**
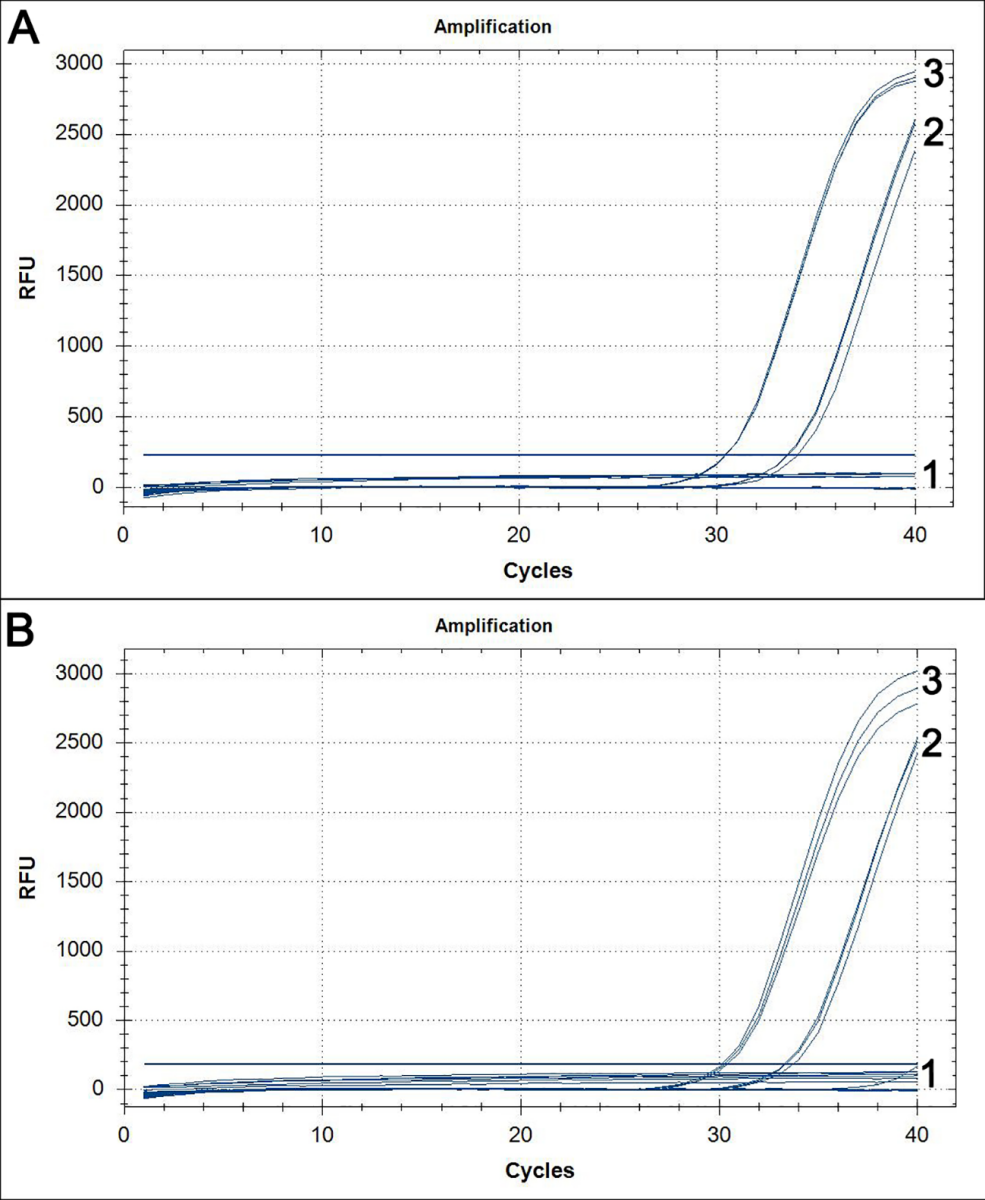
Specificity test of the WYN029GmA qPCR detection method. **(A)** Combination 9, WYN029LB-QF4/QR1/QP2. **(B)** Combination 10, WYN029LB-QF4/QR2/QP2. Curve 1: blank control, negative control, mixed samples of other GM soybeans, mixed samples of GM maize, mixed samples of GM cotton, mixed samples of GM rapeseed, mixed samples of GM rice, and a mixed sample of non-transgenic soybeans. Curve 2: positive control. Curve 3: 1% WYN029GmA.

The experimental results are shown in **Figure 2**. Both primer-probe combinations 9 and 10 passed the specificity test, with the expected amplification curve observed only in WYN029GmA soybean. Based on previous screening results, combination 10 (WYN029LB-QF4/QR2/QP2) showed better stability and was therefore selected as the optimal primer-probe combination for the event-specific detection method of WYN029GmA.

**Figure 3:**
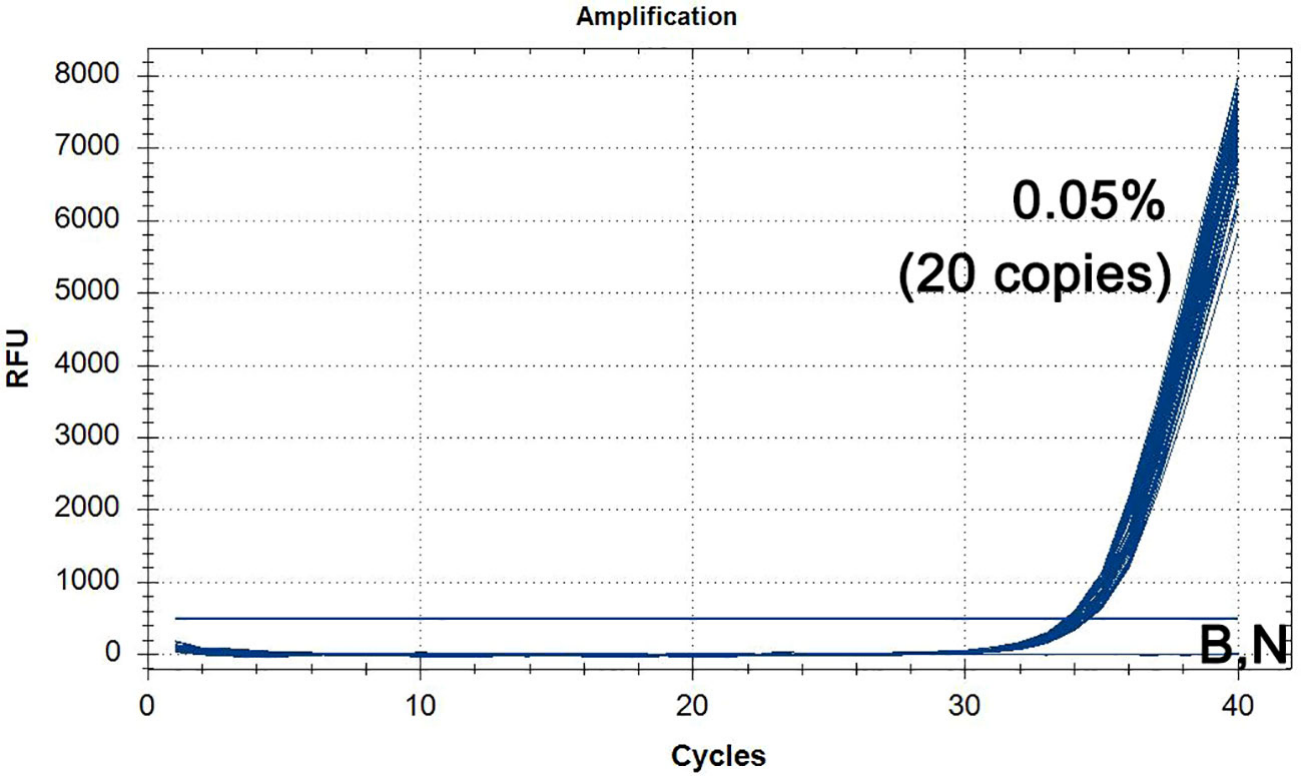
Amplification curves for limit of detection(LOD) determination of the WYN029GmA qPCR detection method. Data from 60 replicate tests. B, blank control; N, negative control.

The combination 10 (WYN029LB-QF4/QR2/QP2) primer-probe sequences are shown in **Table 1**. The amplified product sequence (5’-TACTCGCACTTTTGTTGTTTCAGACCAAGTATTCAGGAACAGGGGCGGctcaatgacaagaagaaaatcttcgtcaacatggtggagcacgacac-3’) is consistent with the sequence presented in **Figure 3** of the published patent (He et al., 2024).

### 3.3 Determination of primer and probe concentrations

In this experiment, four primer concentrations (0.2, 0.4, 0.6, 0.8 µmol/L) were tested. The probe concentration was set at half of the corresponding primer concentration in each case. As shown in **Supplementary Figure 2**, when the primer concentration was ≥ 0.4 µmol/L, no significant differences in Cq values were observed among the treatments. Published detection standards for the soybean endogenous reference gene *Lectin* (Announcement No. 2031-8–2013 of the Ministry of Agriculture in China) and for transgenic events such as Zhonghuang 6106 (Announcement No. 864-16–2024 of the Ministry of Agriculture and Rural Affairs in China) provide reference primer and probe concentrations. Based on these considerations, taking into account the convenience and efficiency of reaction mixture preparation, the final concentrations in the event-specific detection system for WYN029GmA were determined as 0.4 µmol/L for the primers and 0.2 µmol/L for the probe.

### 3.4 LOD

The preliminary test results are presented in **Supplementary Figure 3**, **Supplementary Table 2**. When the template amount was 1 copy, 5 out of 10 reactions were negative, confirming that the copy numbers of the dilution series were approximately accurate. For template amounts of 5, 10, 20, and 40 copies, typical amplification curves were observed in all 10 reactions. For template amounts of ≥10 copies, the Cq values of all reactions were ≤36. Considering the amplification curves, Cq values, and the robustness of the method, the LOD was preliminarily determined to be 0.05% (equivalent to approximately 20 copies of WYN029GmA).

To further validate this LOD, 60 replicate tests were performed on samples containing 0.05% of the WYN029GmA event. All reactions exhibited typical amplification curves (**Figure 3**). Compared with reported event-specific methods, the LOD of this method (0.05%, 20 copies) is comparable to that of CC-2 maize qPCR method (Long et al., 2024) and LD05 maize qPCR methods (Li et al., 2025). Although the DBN8002 soybean qPCR method (Yao et al., 2024) achieved a lower LOD (0.0125%, 5 copies), it did not present data from 60 replicate tests. These results demonstrate that the LOD of the WYN029GmA qPCR detection method is comparable to that of similar methods, and samples containing as low as 0.05% of the WYN029GmA event can be reliably detected (European Network of GMO Laboratories (ENGL), 2015).

### 3.5 Establishment of standard curves

**Figure 4:**
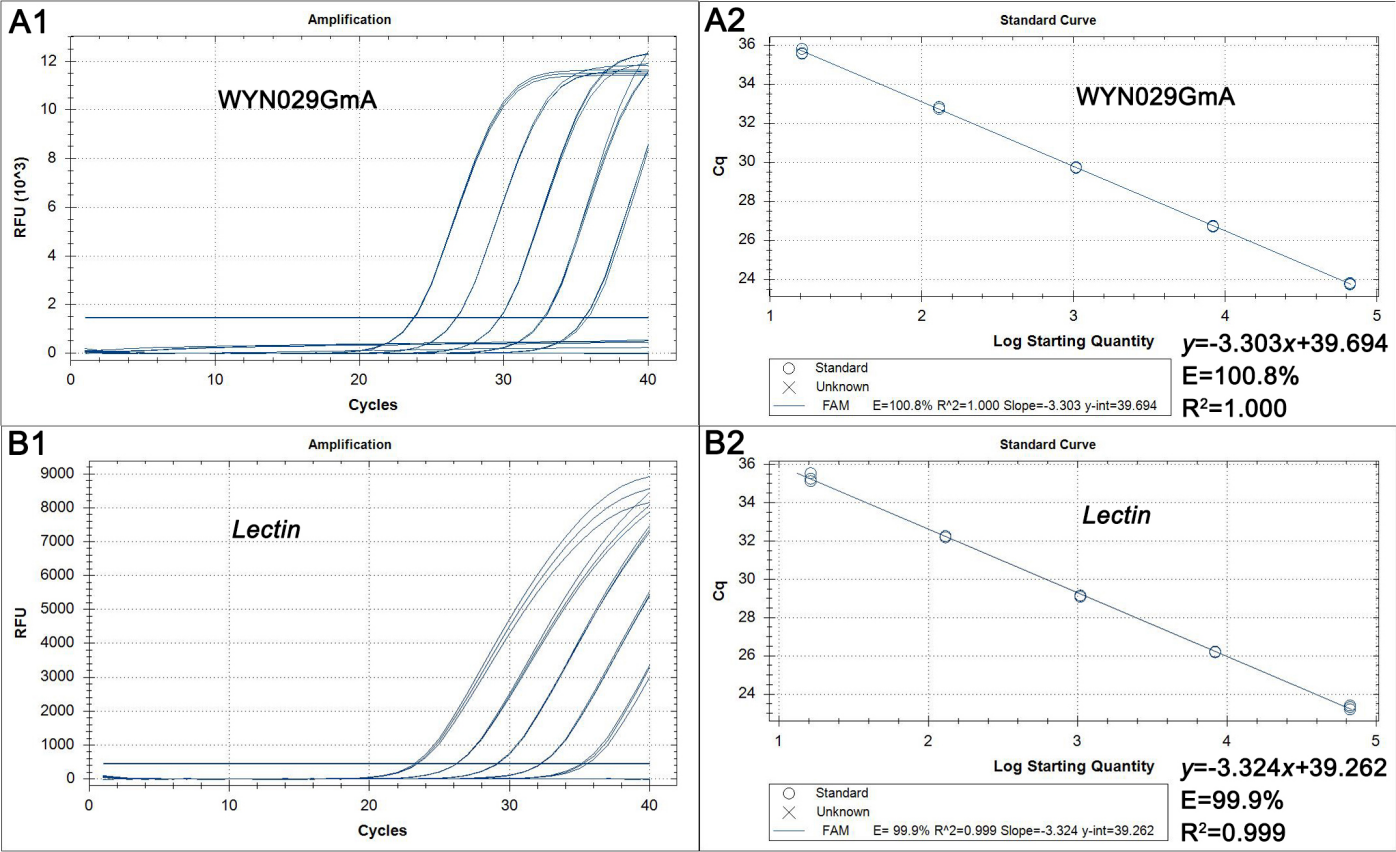
Representative standard curves from the first replicate experiment. **(A1)** Amplification plot for the WYN029GmA event. **(A2)** Standard curve for WYN029GmA event. **(B1)** Amplification plot for the endogenous gene *Lectin*. **(B2)** Standard curve for *Lectin* gene.

The standard curves for the WYN029GmA event and the *Lectin* gene were generated by plotting the Cq values from PCR of standard DNA solutions against the logarithm of the initial template copy number. Three independent replicate experiments were performed. Representative results from the first replicate experiment are shown in **Figure 4**, and the standard curves from the other two replicates are provided in **Supplementary Figure 4**. The slope, coefficient of determination (R²), and PCR amplification efficiency (E) of each curve are summarized in **Supplementary Table 3**. For the WYN029GmA event, the slopes of the standard curves from the three experiments ranged from –3.368 to –3.303, R² values ranged from 0.999 to 1.000, and E values ranged from 98.1% to 100.8%. For the endogenous reference gene *Lectin*, the slopes ranged from –3.324 to –3.316, R² values ranged from 0.998 to 0.999, and E values ranged from 99.9% to 100.2%. All data parameters of the standard curves met the requirements for quantitative GM detection methods (European Network of GMO Laboratories (ENGL), 2015), indicating a good linear relationship between the logarithm of the template copy number and Cq values for the WYN029GmA detection system established in this study.

### 3.6 Trueness and precision

The experimental results are shown in **Table 2**. The relative bias (*biasR*) between the measured mean values and the nominal values of the four samples at different content levels ranged from –2.71% to 7.79%, all within the acceptable limits of ±25%. The relative repeatability standard deviation (*RSDr*) ranged from 3.95% to 10.13%, all below the specified threshold of 25%. These results indicate that both the trueness and precision of the quantitative results met the performance requirements for GMO analytical methods (European Network of GMO Laboratories (ENGL), 2015), confirming that the established qPCR method can accurately quantify the WYN029GmA content in DNA samples.

**Table 2:** Trueness and precision of the qPCR assay for WYN029GmA at different expected values.

Expected value (%)	Experimental value (%)			Mean (%)	*biasR* (%)	*RSDr* (%)
	Subsample 1	Subsample 2	Subsample 3			
5.00	4.99	5.41	5.10	5.17	3.35	4.87
3.00	3.32	3.10	3.28	3.23	7.79	3.95
1.00	0.96	0.90	1.06	0.97	-2.71	10.13
0.10	0.10	0.11	0.10	0.10	3.34	4.63

*biasR*, the relative bias; *RSDr*, the relative repeatability standard deviation.

### 3.7 LOQ

The preliminary experimental results are presented in **Supplementary Figure 5**, **Supplementary Table 4**. When the template amount was 1 copy, 4 out of 10 reactions were negative, confirming that the copy numbers of the dilution series were approximately accurate. For template amounts of 20, 30, 40, and 50 copies, all 10 reactions exhibited typical amplification curves, with *biasR* of 13.23%, 3.63%, -1.09%, and 3.03%, and *RSDr* of 14.50%, 10.33%, 11.34%, and 7.18%, respectively. Based on the above data, when the copy number ratio of the WYN029GmA event was 0.05% or higher (equivalent to approximately 20 copies of WYN029GmA), accurate quantification could be achieved: all 10 reactions were positive, and the results of trueness and precision analyses met the requirements. Nevertheless, to ensure the robustness in quantitative experiments, the LOQ of this method was preliminarily determined to be 0.1% (equivalent to approximately 40 copies of WYN029GmA).

To further validate this LOQ, 15 replicate tests were conducted on samples with a copy number ratio of 0.1%. As shown in **Table 3**, |*biasR*| and the *RSDr* were ≤25%, falling within the acceptable range. Consequently, the LOQ of the qPCR method for the WYN029GmA event was determined to be 0.1%, meeting the requirements of EU standards (European Network of GMO Laboratories (ENGL), 2015).

**Table 3:** Limit of quantification (LOQ) test results of qPCR for WYN029GmA.

Repeat	WYN029GmA(copies)	*Lectin*(copies)	Experimental value (%)
1	40	33310	0.121
2	31	33180	0.095
3	37	33980	0.109
4	34	35980	0.095
5	31	39130	0.080
6	34	38470	0.089
7	37	38330	0.098
8	33	34440	0.097
9	34	34500	0.098
10	38	35250	0.107
11	34	38010	0.088
12	35	36140	0.098
13	31	37680	0.082
14	40	36550	0.109
15	39	35960	0.110
**Mean**	**35**	**36061**	**0.098**
***BiasR* (%)**	**-**	**-**	**-1.597**
***RSDr* (%)**	**-**	**-**	**11.314**

Mean, the average value; *biasR*, the relative bias; *RSDr*, the relative repeatability standard deviation; -, not applicable.

Bold values indicate the mean (average value).

### 3.8 Robustness

The robustness test for the LOD (**Supplementary Figure 6**) demonstrated that consistent detection results could be obtained across different operators and instruments. The robustness test for the LOQ (**Supplementary Table 5**) indicated that varying the operators, reagents, and instruments during detection still yielded trueness and precision values meeting the acceptance criterion of ≤25%, confirming that the quantitative results were accurate and reliable. In summary, the WYN029GmA event-specific qPCR method demonstrated satisfactory robustness.

### 3.9 Collaborative validation of the event-specific qPCR method for WYN029GmA

An interlaboratory collaborative study was conducted to evaluate the performance of the established method under different laboratory conditions. The validation results were consistent with those from intralaboratory assessments. Typical amplification curves were observed in the 1% and 0.05% samples but not in the others, indicating that the method had good specificity and achieved an LOD of 0.05%.

The standard curve parameters for each laboratory are summarized in **Supplementary Table 6**. For the WYN029GmA event, the slope of the standard curves ranged from –3.467 to –3.145, and for the *Lectin* gene, from –3.540 to –3.130.The R² values ranged from 0.984 to 1.000 for WYN029GmA and from 0.987 to 1.000 for *Lectin*, all exceeding the acceptable threshold of 0.98. The E values ranged from 94.300% to 108.000% for WYN029GmA and from 91.640% to 108.700% for *Lectin*, both falling within the permissible range of 90% to 110%. All parameters met the requirements specified in the Announcement No. 2259-5–2015 of the Ministry of Agriculture and Rural Affairs in China.

Based on the test data from eight laboratories on five samples with different concentration levels (three parallel subsamples per level), Cochran’s test and Grubb’s test were conducted according to Chinese National Standard GB/T 6379.2-2004. The statistical analysis results are summarized in **Table 4**. After testing, the data from laboratory 4 at the 5% concentration level were identified as outliers and removed, while the remaining data were considered valid. The overall mean values of the five samples at different concentration levels tested by eight laboratories were 5.28%, 3.22%, 0.98%, 0.50%, and 0.11%, respectively. The *biasR* between the overall mean and the nominal values ranged from –2.250% to 7.458%, all within the acceptable limit of ±25%, indicating high trueness of the method. The *RSDr* ranged from 4.117% to 8.106%, below the specified limit of 25%, while the relative reproducibility standard deviation (*RSD_R_*) ranged from 7.888% to 11.114%, below the specified limit of 35%. The repeatability and reproducibility analysis results demonstrate that the method exhibits good precision. In summary, both trueness and precision met the requirements of the Announcement No. 2259-5–2015 of the Ministry of Agriculture and Rural Affairs in China, confirming that the method has satisfactory accuracy.

**Table 4:** Repeatability and reproducibility for five content levels (expected values from 0.1% to 5.0%).

Statistic	Expected value (%)				
	5.0	3.0	1.0	0.5	0.1
Overall mean of test results (%)	5.279	3.216	0.978	0.497	0.107
*biasR* (%)	5.581	7.194	-2.250	-0.667	7.458
*RSD*_r_ (%)	4.117	4.258	5.753	5.359	8.106
*RSD*_R_ (%)	7.888	8.978	10.489	11.114	9.329

*biasR*, the relative bias; *RSDr*, the relative repeatability standard deviation; *RSD*_R_, the relative reproducibility standard deviation.

The collaborative validation results for the LOQ (**Supplementary Table 7**) showed that the average values obtained from the 0.1% samples across eight laboratories ranged from 0.10% to 0.12%, with an overall mean of 0.107% and a *biasR* of 7.458%. In addition, both the *RSDr* and the *RSD_R_* were below 25%. These results indicate that the LOQ of this method can reach 0.1% (equivalent to approximately 40 copies of WYN029GmA), which is consistent with the intralaboratory results.

### 3.10 Measurement uncertainty of the tested results

**Figure 5:**
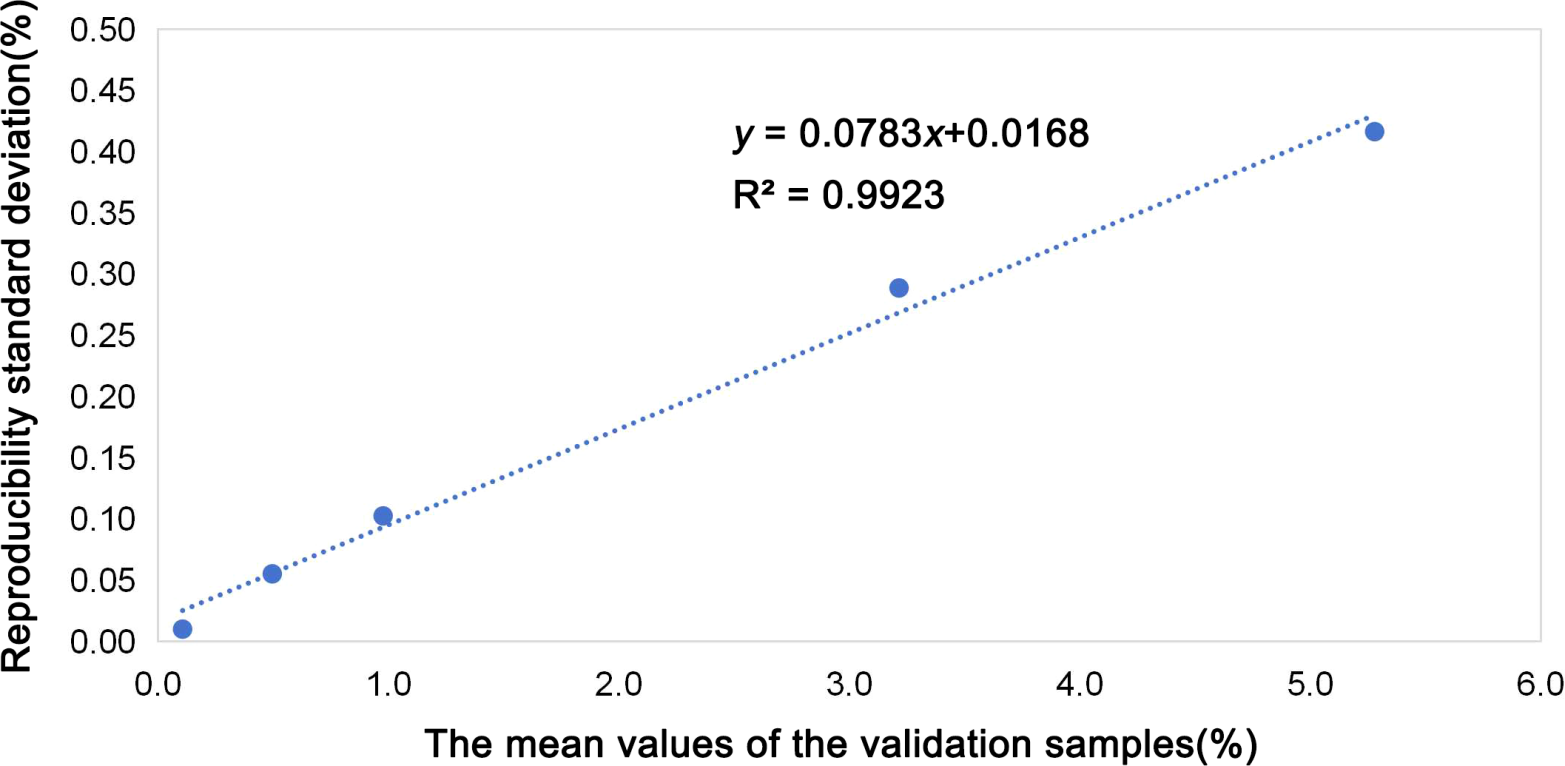
Linear relationship between the reproducibility standard deviation from the interlaboratory validation of the WYN029GmA detection method and the mean detected values of the validation samples.

According to the guidance documents (Trapmann et al., 2007; Standardization ISO/TS 21748:2004), measurement uncertainty (MU) for the quantitative results was estimated. Based on the data in **Table 4**, a regression curve was constructed by plotting the mean values of the validation samples ( $\bar{\bar{c}}$) at each level on the x-axis against the reproducibility standard deviation obtained from the interlaboratory validation on the y-axis, as shown in **Figure 5**. The relative standard uncertainty component (*u_r_*) and the absolute standard uncertainty component (*u_0_*) of the quantitative results were from this regression curve. The regression equation was y=0.0783x+0.0168, where the slope (0.0783) represented *u_r_* and the intercept (0.0168) represented *u_0_*. Laboratories can evaluate the uncertainty of quantitative results using the pre-estimated *u_0_* and *u_r_* derived from the collaborative validation data of this method. The standard uncertainty (*u*) of a sample’s quantitative result was calculated according to the formula $\text{u}=\sqrt{0.0168^{2}+{\left(0.0783\times \bar{\bar{c}}\right)}^{2}}$. The expanded uncertainty (U) was obtained as U = ku, with a coverage factor k=2 at the 95% confidence level.

## 4 Conclusion

In this study, an event-specific detection method for the herbicide-tolerant soybean WYN029GmA based on TaqMan qPCR technology was successfully established, enabling both qualitative and quantitative detection of the WYN029GmA event in soybeans and derived products. Through the screening of 12 sets of primers and probes, the combination WYN029LB-QF4/QR2/QP2 was identified as the optimal choice, exhibiting the best specificity, amplification efficiency, and stability. Under the preferred reaction system, the LOD of this method reached 0.05%, and the LOQ reached 0.1%; the R² values of the standard curves were all ≥0.998, with E values ranging from 98.1% to 100.8%; the *biasR* (–2.71% to 7.79%) and *RSDr* (3.95% to 10.13%) for samples with different contents all met the acceptance criteria: |*biasR*| and *RSDr* ≤ 25%. Furthermore, robustness testing and a collaborative validation involving eight laboratories further confirmed the reliability and repeatability of the method under different conditions. The qPCR method established in this study for WYN029GmA exhibits strong specificity, good repeatability, and high accuracy, which can provide reliable technical support for the detection, monitoring, and standardized application of transgenic components in herbicide-tolerant soybean WYN029GmA.

## Data Availability

The original contributions presented in the study are included in the article/**Supplementary Material**. Further inquiries can be directed to the corresponding author/s.
