# Supplementary material for: Development and validation of an event-specific detection method for WYN029GmA soybean based on TaqMan qPCR
Source: Front Plant Sci. 2026 Jun 12;17:1862064. doi: 10.3389/fpls.2026.1862064 (PMC13303970; doi:10.3389/fpls.2026.1862064)
Supplement: Supplementary file 1 [file Image1.pdf]

frontiers

Supplementary Figure

B.

C.

D.

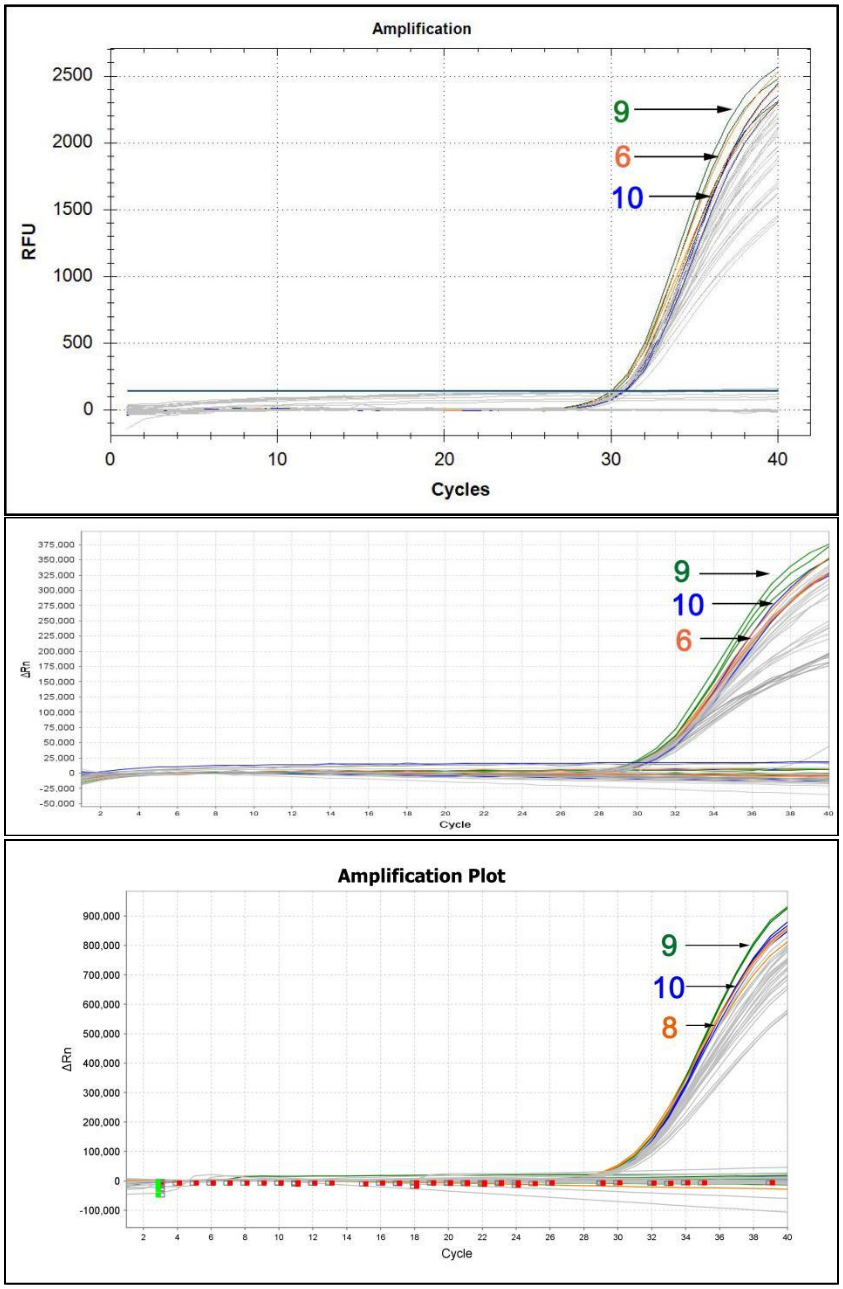

Figure S1. Additional qPCR amplification results of 12 primer/probe combinations of WYN029GmA under different instrument and reagent conditions. (B) Instrument: CFX96; Reagent: Premix Ex Taq™ Probe qPCR. (C) Instrument: StepOnePlus; Reagent: Premix Ex Taq™ Probe qPCR. (D) Instrument: QuantStudio 3; Reagent: TaqMan™ Fast Advanced Master Mix. Curves 9, 6, 10, 8 represent primer/probe combination 9, 6, 10, 8, respectively.

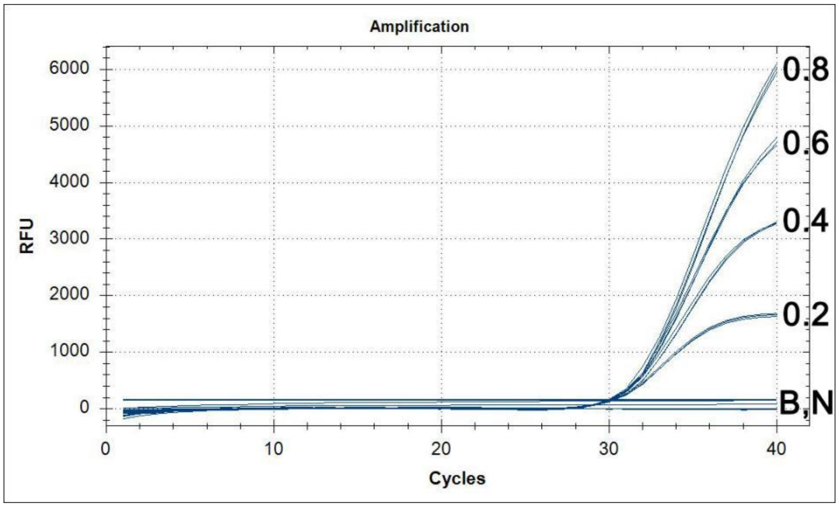

Figure S2. Determination of primer and probe concentrations of WYN029GmA qPCR detection method. Amplification curves for primer concentrations of 0.2, 0.4, 0.6, and 0.8 μmol/L (with probe concentrations at half these values). The legend values (0.2, 0.4, 0.6, 0.8) indicate primer concentrations in μmol/L. B, blank control (no template); N, negative control (non-target template).

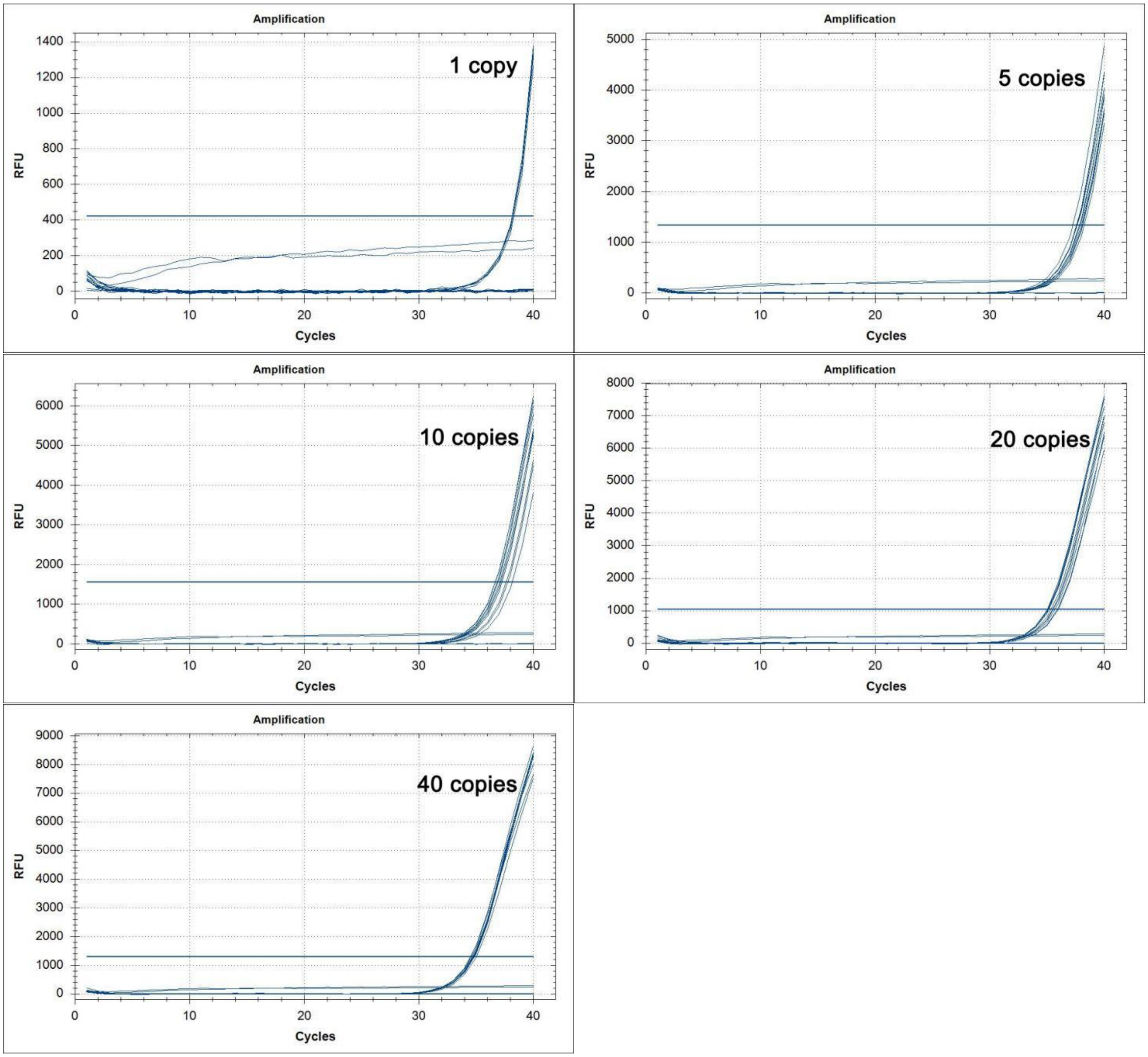

Figure S3. Preliminary test of the LOD of WYN029GmA qPCR detection method. Template amounts of 1, 5, 10, 20, and 40 copies are labeled on the respective panels.

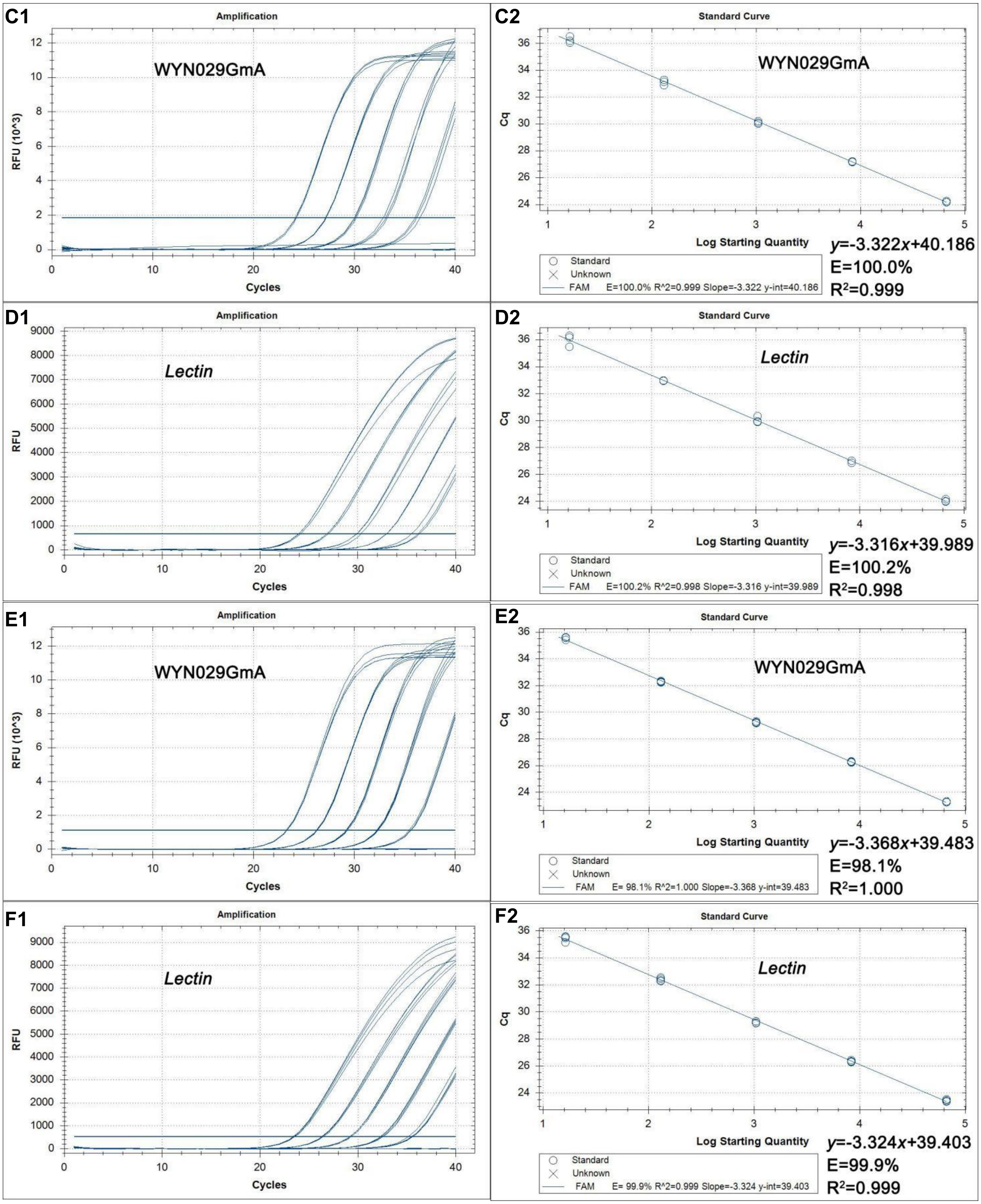

Figure S4. Standard curves from the second and third replicate experiments for the WYN029GmA event and the *Lectin* gene.(C1, C2) Amplification plot (C1) and standard curve (C2) for the WYN029GmA event from the second replicate experiment. (D1, D2) Amplification plot (D1)

and standard curve (D2) for the endogenous gene *Lectin* from the second replicate experiment. (E1, E2) Amplification plot (E1) and standard curve (E2) for the WYN029GmA event from the third replicate experiment. (F1, F2) Amplification plot (F1) and standard curve (F2) for the *Lectin* gene from the third replicate experiment.

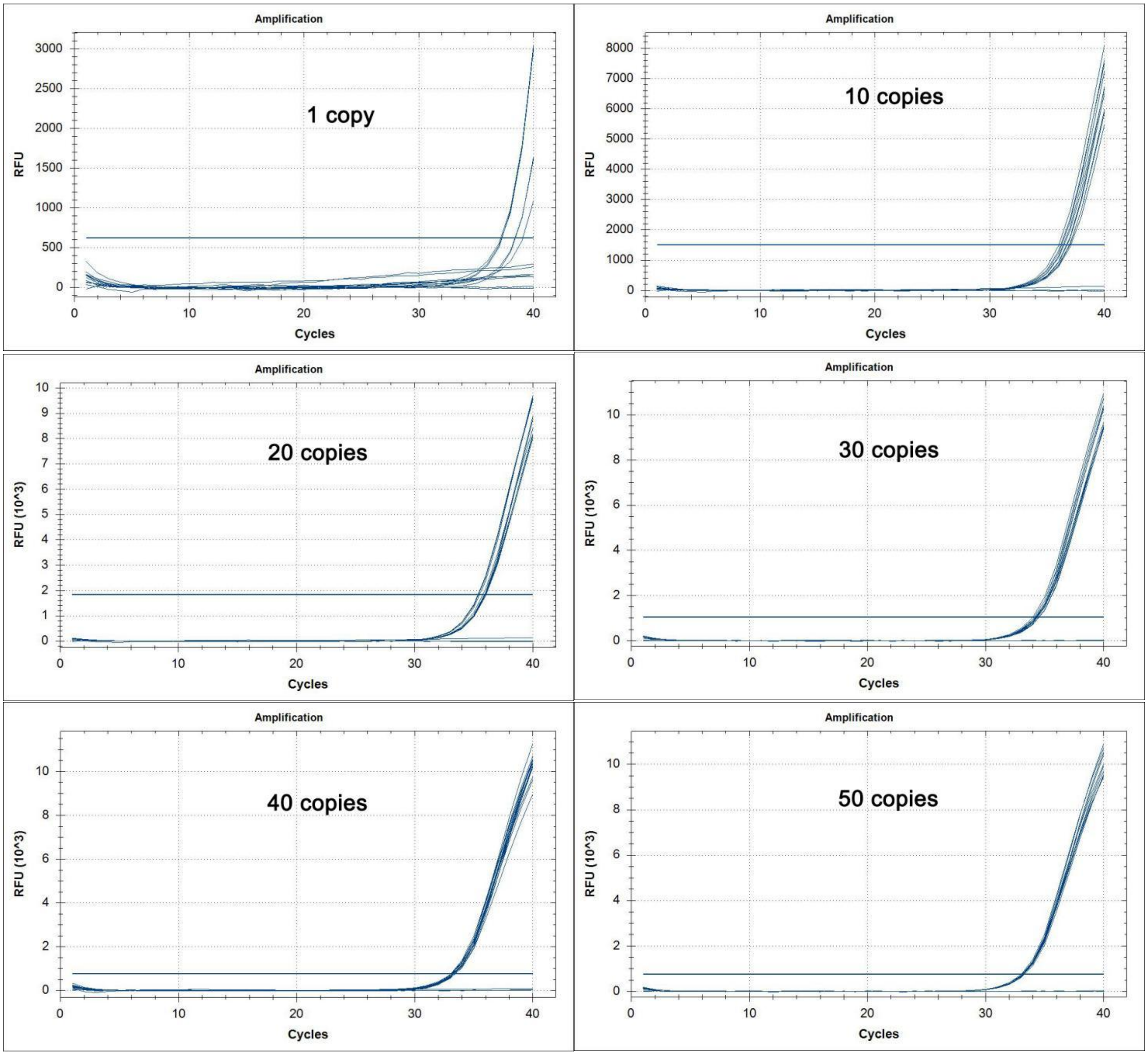

Figure S5. Preliminary test of the LOQ of WYN029GmA qPCR detection method. Template amounts of 1, 10, 20, 30, 40 and 50 copies are labeled on the respective panels.

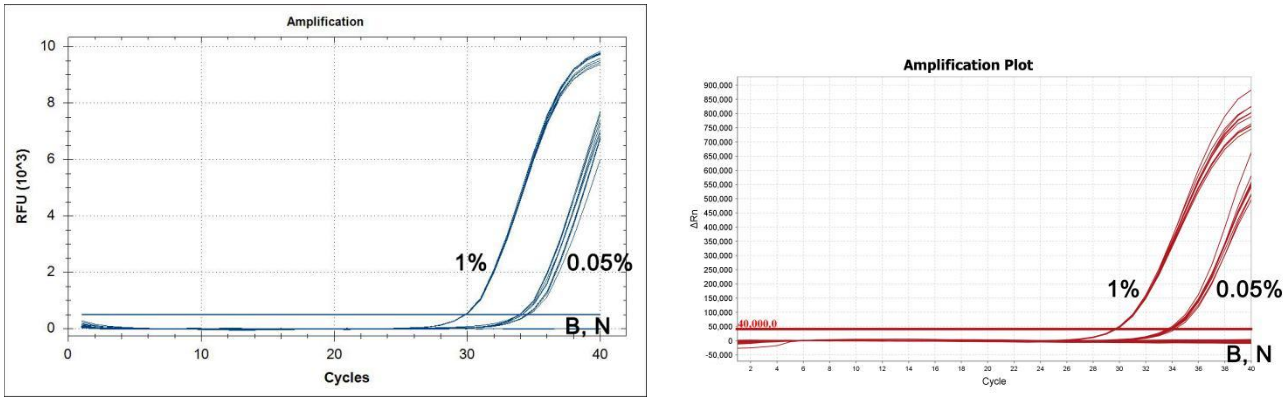

Figure S6. Robustness test of the LOD of WYN029GmA qPCR detection method. (A) Operator 1 using CFX96 instrument; (B) Operator 2 using Quant Studio 3 instrument.
