# Supplementary material for: Development and validation of an event-specific detection method for WYN029GmA soybean based on TaqMan qPCR
Source: Front Plant Sci. 2026 Jun 12;17:1862064. doi: 10.3389/fpls.2026.1862064 (PMC13303970; doi:10.3389/fpls.2026.1862064)
Supplement: Supplementary file 2 [file Table1.docx]

Supplementary Table

Table S1. Primer-probe combinations for qPCR detection of WYN029GmA

| **No.** | **Combination** | **Product size（bp）** | **No.** | **Combination** | **Product size（bp）** |
| --- | --- | --- | --- | --- | --- |
| 1 | WYN029LB-QF1/QR1/QP1 | 163 | 7 | WYN029LB-QF3/QR2/QP2 | 108 |
| 2 | WYN029LB-QF1/QR1/QP2 | 163 | 8 | WYN029LB-QF4/QR1/QP1 | 139 |
| 3 | WYN029LB-QF1/QR2/QP2 | 99 | 9 | WYN029LB-QF4/QR1/QP2 | 139 |
| 4 | WYN029LB-QF2/QR1/QP1 | 117 | 10 | WYN029LB-QF4/QR2/QP2 | 95 |
| 5 | WYN029LB-QF3/QR1/QP1 | 152 | 11 | WYN029RB-QF1/QR1/QP1 | 98 |
| 6 | WYN029LB-QF3/QR1/QP2 | 152 | 12 | WYN029RB-QF2/QR1/QP1 | 172 |

Table S2. Preliminary test results of limit of detection (LOD) of qPCR for WYN029GmA (Cq values)

| **Repeat** | **40 copies** | **20 copies** | **10 copies** | **5 copies** | **1 copy** |
| --- | --- | --- | --- | --- | --- |
| 1 | 32.95 | 33.48 | 35.07 | 35.69 | ND |
| 2 | 33.02 | 34.17 | 34.85 | 35.62 | ND |
| 3 | 32.92 | 34.36 | 34.48 | 35.95 | 38.20 |
| 4 | 32.70 | 33.61 | 35.56 | 36.05 | 38.07 |
| 5 | 33.17 | 33.85 | 34.65 | 35.97 | 38.08 |
| 6 | 32.68 | 33.54 | 34.74 | 36.15 | ND |
| 7 | 32.92 | 34.05 | 35.41 | 35.31 | ND |
| 8 | 32.91 | 33.56 | 34.42 | 36.32 | 38.13 |
| 9 | 32.75 | 34.41 | 35.01 | 36.17 | 38.05 |
| 10 | 32.94 | 34.00 | 36.00 | 35.69 | ND |
| Mean ± SD | 32.90±0.15 | 33.90±0.35 | 35.02±0.51 | 35.89±0.31 | - |
| Positive/Total | 10/10 | 10/10 | 10/10 | 10/10 | 5/10 |

Note: ND, not detected; -, not applicable.

Table S3. Parameters of standard curves for WYN029GmA and *Lectin*

| **Repeat** | **WYN029GmA** | | | |  | ***Lectin*** | | | |
| --- | --- | --- | --- | --- | --- | --- | --- | --- | --- |
|  | **R^2^** | **Slope** | **Intercept** | **Amplification efficiency (%)** |  | **R^2^** | **Slope** | **Intercept** | **Amplification efficiency (%)** |
| Rep1 | 1.000 | -3.303 | 39.694 | 100.8 |  | 0.999 | -3.324 | 39.262 | 99.9 |
| Rep2 | 0.999 | -3.322 | 40.186 | 100.0 |  | 0.998 | -3.316 | 39.989 | 100.2 |
| Rep3 | 1.000 | -3.368 | 39.483 | 98.1 |  | 0.999 | -3.324 | 39.403 | 99.9 |
| Mean | 1.000 | -3.331 | 39.788 | 99.6 |  | 0.999 | -3.321 | 39.551 | 100.0 |

Table S4A. Preliminary test results: quantitative data for LOQ estimation of WYN029GmA qPCR method

| **Expected value (%)** | **Experimental value(%)** | | | | | | | | | | **Mean (%)** | ***biasR***（%） | ***RSD_r_***（%） |
| --- | --- | --- | --- | --- | --- | --- | --- | --- | --- | --- | --- | --- | --- |
|  | 1 | 2 | 3 | 4 | 5 | 6 | 7 | 8 | 9 | 10 |  |  |  |
| 0.125  （50 copies） | 0.140 | 0.129 | 0.127 | 0.124 | 0.137 | 0.113 | 0.141 | 0.124 | 0.118 | 0.135 | 0.129 | 3.027 | 7.184 |
| 0.100  （40 copies） | 0.121 | 0.095 | 0.109 | 0.095 | 0.080 | 0.089 | 0.098 | 0.097 | 0.098 | 0.107 | 0.099 | -1.086 | 11.337 |
| 0.075  （30 copies） | 0.076 | 0.077 | 0.067 | 0.076 | 0.077 | 0.067 | 0.093 | 0.081 | 0.076 | 0.087 | 0.078 | 3.629 | 10.327 |
| 0.050  （20 copies） | 0.057 | 0.047 | 0.048 | 0.049 | 0.066 | 0.068 | 0.052 | 0.065 | 0.062 | 0.051 | 0.057 | 13.227 | 14.504 |

Note: *biasR*, the relative bias; *RSD_r_*, the relative repeatability standard deviation.

Table S4B. Cq values for dilutions below the LOQ in the preliminary test (same experiment as Table S4A)

| **Expected value (%)** | ***Cq* value** | | | | | | | | | |
| --- | --- | --- | --- | --- | --- | --- | --- | --- | --- | --- |
|  | 1 | 2 | 3 | 4 | 5 | 6 | 7 | 8 | 9 | 10 |
| 0.025（10 copies） | 36.14 | 35.89 | 36.3 | 36.26 | 36.37 | 35.81 | 35.54 | 36.1 | 35.65 | 36.14 |
| 0.0025（1 copy） | 37.26 | ND | ND | 39.07 | ND | 38.39 | 37.16 | 37.23 | ND | 37.26 |

Note: ND, not detected; -, not applicable.

Table S5. Robustness assessment of WYN029GmA qPCR assay at the LOQ (0.1%)

| **Variation factor** | **Experimental value(%)** | | | **Mean** | ***BiasR* (%)** | ***RSD_r_* (%)** |
| --- | --- | --- | --- | --- | --- | --- |
|  | **Subsample 1** | **Subsample 2** | **Subsample 3** |  |  |  |
| Different operator | 0.087 | 0.091 | 0.088 | 0.089 | -11.352 | 3.022 |
| Different reagent | 0.099 | 0.093 | 0.090 | 0.094 | -6.044 | 5.960 |
| Different instrument | 0.115 | 0.100 | 0.100 | 0.105 | 5.133 | 8.691 |

Note: The expected value was 0.1%. For each variation factor, one experiment was performed with three subsamples, and each subsample was measured in three technical replicates. The values shown are the means of the three replicates. Mean, *biasR* (the relative bias), and *RSDr* (the relative repeatability standard deviation) were calculated from the three subsample means (n=3).

Table S6. Summary of standard curve parameters for WYN029GmA and *Lectin* across eight laboratories

| **Laboratory** | **No.** | **WYN029GmA** | | |  | ***Lectin*** | | |
| --- | --- | --- | --- | --- | --- | --- | --- | --- |
|  |  | ***R*^2^** | **Slope** | **Amplification efficiency (%)** |  | ***R*^2^** | **Slope** | **Amplification efficiency (%)** |
| **Lab 1** | 1 | 0.999 | -3.467 | 94.300 |  | 0.999 | -3.476 | 94.000 |
|  | 2 | 0.998 | -3.467 | 94.300 |  | 1.000 | -3.468 | 94.200 |
|  | 3 | 0.999 | -3.414 | 96.300 |  | 0.999 | -3.426 | 95.800 |
| **Lab 2** | 1 | 0.999 | -3.399 | 96.889 |  | 0.997 | -3.462 | 94.462 |
|  | 2 | 0.998 | -3.374 | 97.868 |  | 0.999 | -3.375 | 97.828 |
|  | 3 | 0.998 | -3.361 | 98.391 |  | 0.996 | -3.389 | 97.291 |
| **Lab 3** | 1 | 0.998 | -3.221 | 104.400 |  | 0.998 | -3.209 | 104.900 |
|  | 2 | 0.999 | -3.145 | 108.000 |  | 0.999 | -3.130 | 108.700 |
|  | 3 | 0.999 | -3.158 | 107.300 |  | 0.998 | -3.257 | 102.800 |
| **Lab 4** | 1 | 0.992 | -3.438 | 95.380 |  | 0.996 | -3.540 | 91.640 |
|  | 2 | 0.984 | -3.452 | 94.830 |  | 0.989 | -3.358 | 98.510 |
|  | 3 | 0.992 | -3.408 | 96.510 |  | 0.987 | -3.445 | 95.120 |
| **Lab 5** | 1 | 0.999 | -3.356 | 98.593 |  | 1.000 | -3.297 | 101.046 |
|  | 2 | 0.999 | -3.260 | 102.654 |  | 0.999 | -3.280 | 101.795 |
|  | 3 | 1.000 | -3.274 | 102.035 |  | 0.999 | -3.288 | 101.457 |
| **Lab 6** | 1 | 0.998 | -3.403 | 96.700 |  | 0.999 | -3.264 | 102.500 |
|  | 2 | 0.998 | -3.323 | 100.000 |  | 0.998 | -3.182 | 106.200 |
|  | 3 | 0.998 | -3.409 | 96.500 |  | 0.998 | -3.275 | 102.000 |
| **Lab 7** | 1 | 0.997 | -3.306 | 100.700 |  | 0.999 | -3.444 | 95.200 |
|  | 2 | 0.999 | -3.335 | 99.400 |  | 0.999 | -3.415 | 96.200 |
|  | 3 | 0.997 | -3.345 | 99.000 |  | 0.999 | -3.436 | 95.400 |
| **Lab 8** | 1 | 0.995 | -3.369 | 98.064 |  | 0.994 | -3.337 | 99.358 |
|  | 2 | 0.998 | -3.402 | 96.752 |  | 0.996 | -3.402 | 96.779 |
|  | 3 | 0.996 | -3.437 | 95.412 |  | 0.998 | -3.393 | 97.133 |

Table S7. Interlaboratory reproducibility of the WYN029GmA qPCR assay at the LOQ (0.1%)

| **Subsample No.** | **Lab1** | **Lab2** | **Lab3** | **Lab4** | **Lab5** | **Lab6** | **Lab7** | **Lab8** | **Overall mean** | ***Bias*R（%）** | ***RSD*_r_（%）** | ***RSD*_R_（%）** |
| --- | --- | --- | --- | --- | --- | --- | --- | --- | --- | --- | --- | --- |
| Subsample 1 | 0.12 | 0.09 | 0.11 | 0.12 | 0.09 | 0.10 | 0.12 | 0.11 | 0.107 | 7.458 | 8.106 | 9.329 |
| Subsample 2 | 0.10 | 0.12 | 0.11 | 0.11 | 0.09 | 0.10 | 0.12 | 0.11 |  |  |  |  |
| Subsample 3 | 0.10 | 0.11 | 0.11 | 0.12 | 0.11 | 0.10 | 0.11 | 0.10 |  |  |  |  |
| Lab mean | 0.11 | 0.11 | 0.11 | 0.12 | 0.10 | 0.10 | 0.12 | 0.11 |  |  |  |  |
| *Sr*(%) | 11.08 | 16.61 | 0.00 | 5.06 | 12.22 | 0.00 | 5.06 | 6.11 |  |  |  |  |

Note: The expected value was 0.1%. *S_r_*, relative standard deviation (coefficient of variation) of the three subsample means within a laboratory, calculated using the range method: *S_r_*=(*Range*/1.693)/*Lab mean*×100%. *biasR*, the relative bias; *RSD_r_*, the relative repeatability standard deviation; *RSD_R_*, the relative reproducibility standard deviation.
